# Supplementary material for: Bone Metastases of Diverse Primary Origin Frequently Express the VDR (Vitamin D Receptor) and CYP24A1
Source: J Clin Med. 2022 Nov 3;11(21):6537. doi: 10.3390/jcm11216537 (PMC9658790; doi:10.3390/jcm11216537)

Supplementary Materials

Evaluation was conducted using the Immunoreactive score (IRS) that was previously described by Remmele and Stegner (IRS):

A (percentage of positive cells)	B (intensity of staining)	IRS Score (multiplication of A and B)
0 = no positive cells	0 = no color reaction	0–1 = negative
1 = <10% positive cells	1 = mild reaction	2–3 = mild
2 = 10–50% positive cells	2 = moderate reaction	4–8 = moderate
3 = 51–80% positive cells	3 = intense reaction	9–12 = strongly positive
4 = >80% positive cells	**IRS-score = A∗B = 0–12**	

We added three representative images of bone metastases as examples to illustrate the evaluation process. The nuclear and cytoplasmatic levels were rated separately. All samples were scored independently and blindly by three observers and afterwards the average IRS-Score was calculated. Subsequently, patients were dichotomised into two (high vs. low) groups of bone metastases protein expression. The threshold value was set at an IRS of 5.

1. Example (Bone metastasis secondary to prostate cancer, also used in Figure 1A): High nuclear VDR expression (for example: red arrow) but low cytoplasmatic VDR expression (for example: blue arrow). One observer rated the nuclear VDR expression with an IRS of 8, the other two with 12. Consequently, it resulted in an average IRS of 10.66 and thus a high nuclear VDR expression. Comparatively, the VDR expression in the cytoplasm was rated with an IRS of 1.66 in this patient, therefore the VDR expression in the cytoplasm was rated as low.

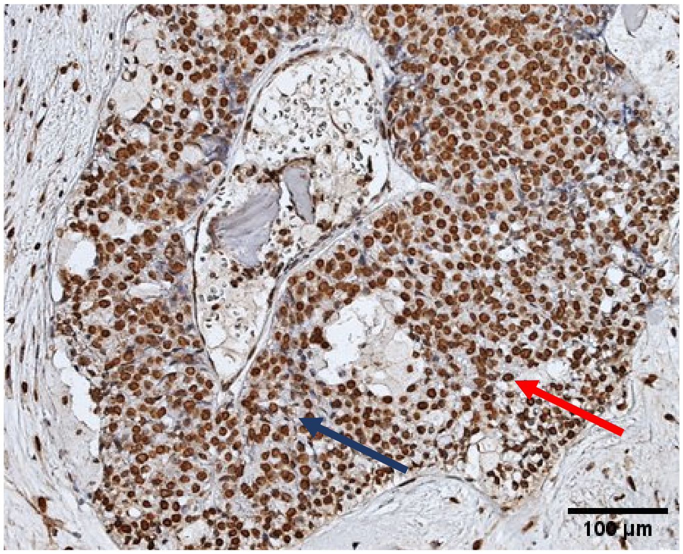

2. Example (Bone metastasis secondary to oesophagus cancer, also used in Figure 1A): Compared to example 1, a higher VDR expression in the cytoplasm is demonstrated here (blue arrow, average IRS 8.66) in addition to a high nuclear VDR expression.

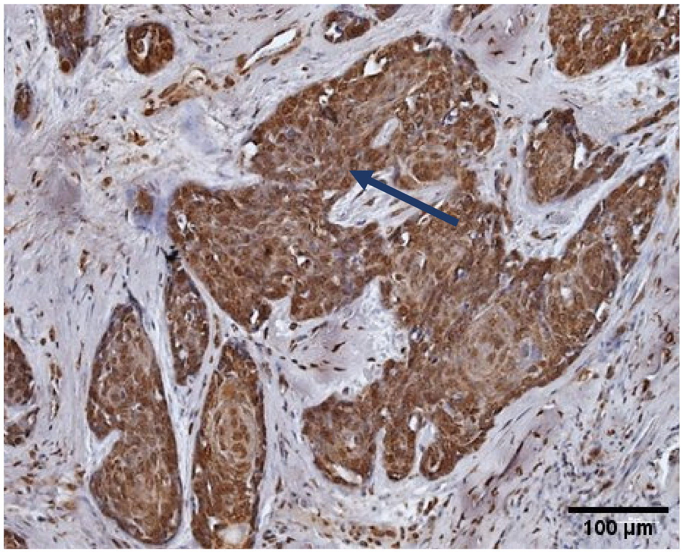

3. Example (Bone metastasis secondary to renal cancer): Compared to example 1 and 2, neither the nuclei nor the cytoplasm demonstrate a high VDR expression

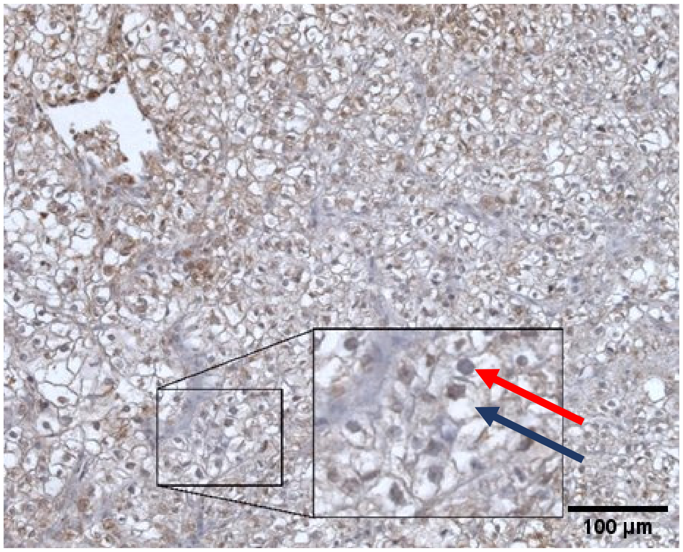

Supplement: Supplementary file 1 [file jcm-11-06537-s001.zip › jcm-1954409-supplementary.pdf]
